# Liver immune tolerance and regulated cell death in transplantation and hepatocellular carcinoma: shared mechanisms, divergent outcomes

**DOI:** 10.3389/fcell.2026.2002295

**Published:** 2026-10-29

**Authors:** Yuerui Wang, Hesong Zhang, Fangxiao Dong, Haomiao Xu, Fei Huang, Bin Jia

**Affiliations:** 1 Shanghai Jiao Tong University School of Medicine, Shanghai, China; 2 Department of Hepatobiliary Surgery, Wuhu Second People’s Hospital, Wuhu, Anhui, China; 3 Suzhou TCM Hospital Affiliated to Nanjing University of Chinese Medicine, Suzhou, Jiangsu, China; 4 Department of Gastroenterology, Affiliated Hospital of Shandong University of Traditional Chinese Medicine, Jinan, Shandong, China

**Keywords:** hepatocellular carcinoma, immune checkpoints, liver immune tolerance, liver transplantation, precision immune homeostasis, regulated cell death, spatial omics, tumor microenvironment

## Abstract

The liver constantly encounters gut-derived antigens, microbial molecules, and food antigens. This environment has shaped a tissue-specific tolerance network centered on tolerogenic antigen presentation, immune-checkpoint activation, regulatory cell expansion, and metabolic suppression. This feature protects the liver during prolonged inflammation and gives it an immunological identity that most solid organs lack. Regulated cell death (RCD) and dead-cell clearance form a critical mechanistic layer within this network. Efficient efferocytosis of apoptotic cells reinforces resolution and tolerance, whereas necroptosis, pyroptosis, and ferroptosis disrupt cell membranes and release danger signals that reshape myeloid activation, T-cell function, and the tumor microenvironment. In liver transplantation, this tolerogenic tendency produces donor-specific hyporesponsiveness, allowing a selected subset of stable recipients to stop immunosuppression while maintaining graft function—a state called operational tolerance. In hepatocellular carcinoma (HCC), tumors exploit the same immunosuppressive axes to block anti-tumor T-cell infiltration and effector function. Anti-PD-L1 therapy combined with anti-angiogenic treatment improves survival, demonstrating that partial disruption of tumor immunosuppression is therapeutically effective. Spatial transcriptomics and single-cell sequencing reveal that SPP1-positive macrophages and cancer-associated fibroblasts form a tumor immune barrier that excludes lymphocytes, showing that hepatic tolerance is organized in space. This duality becomes most dangerous in HCC recurrence after liver transplantation, where immune checkpoint inhibitors boost anti-tumor immunity but also raise the risk of acute rejection, graft failure, and death. Unlike reviews focused on hepatic immune tolerance, regulated cell death, or HCC immunobiology in isolation, this review integrates these processes across the physiological liver, transplantation, HCC, and post-transplant recurrence. We define this coordinated hyporesponsive state as the liver immune tolerance program (ITP), generated by cellular, molecular, spatial, metabolic, and cell-death-related networks. Rather than treating these processes separately, we position RCD and dead-cell clearance as a “death-to-immunity axis,” systematically compare ITP remodeling in the physiological liver, transplantation, and HCC, and use post-transplant HCC recurrence as a clinical crossover setting in which graft protection and antitumor immune surveillance become therapeutic considerations. On this basis, we propose precision immune homeostasis: rather than choosing between immunosuppression and immune activation, immune status should be adjusted to an individualized range that protects the graft while preserving anti-tumor immune surveillance.

## 1 Introduction: from physiological liver tolerance to the paradox of disease-associated tolerance

The immunological task of the liver is inherently contradictory: it needs to continuously receive and process gut-derived pathogen-associated signals via the portal vein, but must avoid damage from inflammation against harmless dietary antigens and components of the commensal microbiota. Thus, “selective hyporesponsiveness” rather than “absolute immune silence” more accurately captures the nature of hepatic immune homeostasis (Kubes and Jenne, 2018). The unique low-shear environment of the hepatic sinusoids, an abundance of resident macrophages, and specialised liver sinusoidal endothelial cells (LSECs) extend the contact time between immune cells and antigen-presenting cells, thus continuously strengthening peripheral tolerance at the tissue level (Kubes and Jenne, 2018; Shetty et al., 2018). LSECs are efficient at internalising circulating antigens but have relatively weak costimulatory capacity; under certain circumstances, they can cause functional hyporesponsiveness, deletion or tolerogenic differentiation of CD8^+^ T cells. Thus, they are the walls of blood vessels and immune ‘gatekeepers’ (Shetty et al., 2018). Kupffer cells clear microbial products, produce immunoregulatory factors such as IL-10, and work with LSECs and hepatic stellate cells to maintain an equilibrium of inflammatory response and tolerance (Araujo David et al., 2026). These features help explain an important observation: the liver exhibits a relatively strong intrinsic capacity for tolerance among solid-organ transplants, yet the same tissue environment may also confer an inherent advantage to tumor cells seeking to evade immune clearance (Kubes and Jenne, 2018; Shetty et al., 2018; Araujo David et al., 2026; Gottwick et al., 2022).

Cell death is a primary cause of this imbalance and not merely a result of damage to the tissue. The immunological significance of a dying cell depends on the execution program, the type of affected cell, the kinetics of membrane disruption and the efficiency of corpse clearance. Apoptotic cells that are rapidly removed by efferocytosis generally promote resolution, but accidental necrosis and regulated lytic programs such as necroptosis, pyroptosis, and ferroptosis can expose or release damage-associated signals that enhance innate immune activation. At the same time, therapy-induced tumour-cell death can be immunogenic by releasing antigens and activating T cells. Therefore, in liver disease, the programmes of cell death and dead-cell clearance should be regarded as essential parts of ITP rather than as its consequences (Galluzzi et al., 2018; Doran et al., 2020; Shi et al., 2024; Galluzzi et al., 2020).

Extensive observation of liver transplantation has identified an operating tolerance that is relatively rare among recipients and not a natural outcome for all patients (Benítez et al., 2013). Early prospective immunosuppression-withdrawal trials in adults showed that some rigorously selected, long-term stable recipients could maintain normal graft function after complete drug discontinuation, supporting the strategy of actively identifying tolerant recipients rather than applying lifelong, uniform-intensity immunosuppression (Benítez et al., 2013). Pediatric recipients, especially those of living-related donor grafts, have also shown good results in successful withdrawal: a typical multicenter study found that a considerable proportion remained stable after gradual tapering and discontinuation, and subsequent larger studies have once again confirmed the existence of identifiable tolerant subgroups among children (Feng et al., 2012; Feng et al., 2021). Although recent in-depth histological follow-up has shown that the normal liver biochemistry may overestimate actual immunological tolerance, subclinical inflammation, interface changes and fibrosis can still occur in apparently stable grafts (Tanimine et al., 2025). In a 2026 international multicenter study, protocolised withdrawal was further shown to be possible for an extended period in highly selected patients; however, success should be evaluated based on multi-dimensional clinical, histological and immunological criteria rather than simply on the absence of medication (Angelico et al., 2026). Therefore, the main scientific questions in hepatic tolerance have moved from whether tolerance exists to which cellular and molecular networks maintain it, how tolerant recipients can be identified, when tolerance can be safely enhanced, and when it needs to be selectively disrupted in the context of cancer (Gottwick et al., 2022; Tanimine et al., 2025; Angelico et al., 2026).

Although previous research has studied hepatic immune tolerance (Ronca et al., 2025), regulated cell death (Hirao et al., 2025), tumour immune evasion (Chen et al., 2023) and transplantation immunology (Prosser et al., 2025) separately, an all-encompassing system linking these processes in physiological liver homeostasis, liver transplantation, HCC and post-transplant HCC recurrence has not been established. Therefore, the three factors mentioned above will not be considered independent causes in this paper; instead, their causal relationships and different applications will be explored. First, the tissue-level framework of the liver immune tolerance programme (ITP) is used to integrate cellular, molecular, metabolic, spatial, and cell-death related mechanisms, and immune tolerance is not defined by a single checkpoint or cell population (Ronca et al., 2025; Gao et al., 2026; Crispe, 2014). Secondly, we position regulated cell death and dead-cell clearance as a “death-to-immunity axis”, and change the same tissue environment to induce resolution and tolerance, sterile inflammation, or anti-tumor immunity depending on the type of cell, location, time course and clearance mechanism (Kroemer, 2016; Zhang et al., 2026; Li et al., 2020). Third, we use post-transplant HCC recurrence as a clinical crossover setting to investigate the combined effects of graft protection and tumour immune surveillance (Li et al., 2020; Yamada et al., 2026). This framework is for readers engaged in research at the intersection of liver transplantation, tumour immunology, cell-death biology and translational hepatology (Figure 1) (Hirao et al., 2025).

**FIGURE 1:**
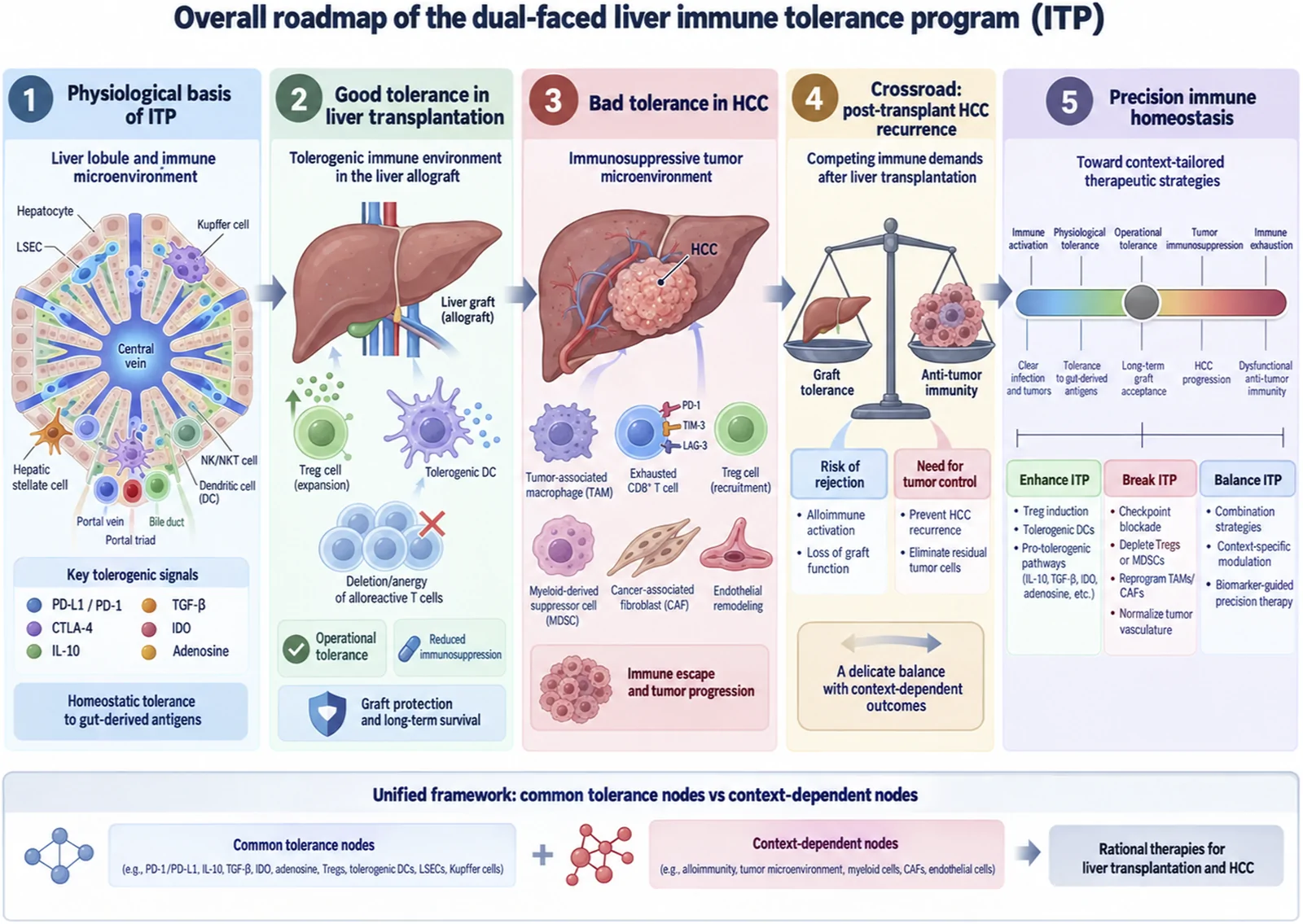
Overall roadmap of the duality of the liver immune tolerance program (ITP).

## 2 Physiological basis of the liver immune tolerance program

To create a universal language for transplantation and cancer, we present the ITP (Organised Hyporesponsive Network); this network involves the cooperation of liver-resident cells, circulating immune cells, inhibitory molecules, metabolic resources and spatial architecture, and is not attributable to a single cell type or checkpoint pathway (Kubes and Jenne, 2018; Shetty et al., 2018; Araujo David et al., 2026; Gottwick et al., 2022). Kupffer cells in the steady-state liver first detect and eliminate microbial products that reach the portal vein, and at the same time, they can suppress inflammatory amplification through signals such as IL-10; thus, their functions do not adhere to a fixed M1/M2 division but rather change along a microenvironment-dependent continuum (Kubes and Jenne, 2018; Araujo David et al., 2026). Efficiently endocytose, cross-present antigens, and present them with low co-stimulation to induce anergy or exhaustion-like states in T cells that enter the liver; additionally, the inhibitory axis of PD-L1 further increases the local threshold for T cell activation (Shetty et al., 2018; Kremer et al., 2024; Henriques-Pons et al., 2024; Olguín-Contreras et al., 2026). Beyond their roles in matrix homeostasis and fibrosis, hepatic stellate cells can regulate immune-cell migration and activation through cytokines, chemokines, and extracellular-matrix remodeling, placing them at the intersection of metabolism, tissue repair, and immune regulation (Kubes and Jenne, 2018). Dendritic cells, NK/NKT cells, regulatory T cells (Tregs), myeloid-derived suppressor cells and regulatory B cells also modulate the local immune response to favour either clearance or tolerance through modifications in antigen-presentation efficiency, cytotoxic thresholds and cytokine environment (Kubes and Jenne, 2018; Gottwick et al., 2022).

Inhibitory receptors at the molecular level, such as PD-1/PD-L1, CTLA-4, LAG-3 and TIM-3, are not independent regulators; together with IL-10, TGF-β, IDO-related tryptophan metabolism, arginine metabolism and the CD39/CD73-adenosine pathway, they form a cooperative suppressive network (Kubes and Jenne, 2018; Gottwick et al., 2022). This redundancy means that blockade of a single node often restores effector immunity only partially, which helps explain why HCC immunotherapy often requires antiangiogenic combinations, dual-checkpoint blockade, or other microenvironmental interventions to achieve higher response rates and more durable clinical benefit (Finn et al., 2020). The ITP is also relatively spatially non-uniform. Differences in oxygen tension, nutrient supply, and cell composition among the portal tract, hepatic sinusoid, and pericentral regions can lead to different functional states of the same type of immune cell in adjacent microenvironments (Kubes and Jenne, 2018; Shetty et al., 2018). The “degree of tolerance” in the system should be regarded as a continuous variable that includes immune activation, physiological tolerance, operational tolerance after transplantation, tumour-associated immunosuppression and profound immune exhaustion, etc., and not a binary state of tolerant/non-tolerant (Kubes and Jenne, 2018; Gottwick et al., 2022). A strength of the continuum model is that the same molecule can have different therapeutic effects under different conditions; for instance, augmenting PD-L1-inhibitory therapy may improve the success of organ transplantation by suppressing tumour-specific T-cell function in hepatocellular carcinoma (HCC) (Gottwick et al., 2022; Finn et al., 2020).

## 3 Regulated cell death as a mechanistic bridge between liver injury and immune reprogramming

Cell death is not only the end result of liver injury; it is also an information-generating phenomenon that determines whether the ITP will resolve, lead to sterile inflammation and graft immunogenicity, or promote antitumor immunity. Currently, the terms accidental cell death and regulated cell death (RCD) are different; RCD is controlled by genetically encoded molecular machinery and can thus be pharmacologically modulated (Galluzzi et al., 2018). The immune consequences of cell death in the liver depend on the type of dying cell, the execution pathway, the kinetics and spatial scale of membrane disruption, and the efficiency of clearance for dead cells. Therefore, the same numerical loss of cells may lead to different immune effects in different cases.

RCD-related immune effects are also varied in experimental and clinical settings (Hu et al., 2025; Naylor et al., 2022). Findings in murine models cannot be assumed to reflect the biology of human liver disease, as there are many differences in cellular composition, disease duration, treatment exposure and tissue structure (Khan et al., 2017). The root cause of HCC is unknown; therefore, it is unclear whether HBV-, HCV-, or metabolic diseases are associated with inflammation and metabolism in RCD (Zhang, 2026). Immunosuppressive therapy in transplanted tissues can both increase the vulnerability of specific cell populations to cell death pathways and alter the immune response to damaged cells (Lim et al., 2015). Therefore, the extrapolated mechanistic results may not apply to all disease situations. Pathway-associated biomarkers, such as caspase activation, RIPK3/MLKL phosphorylation, gasdermin cleavage or lipid-peroxidation signatures, can indicate the activation of a pathway but do not necessarily signify actual cell death (Hu et al., 2021). The assignments of RCDs should incorporate molecular, morphological and functional data to reduce dependence on a single biomarker where possible (Hu et al., 2021).

Apoptosis generally involves caspase activation, cellular breakdown and maintenance of plasma membrane integrity prior to phagocytosis. Exposure of phosphatidylserine and other “eat-me” signals promotes efferocytosis by Kupffer cells and other macrophages via receptors such as MerTK and TIM4. Effective efferocytosis prevents secondary necrosis and can reprogram phagocytes into a pro-resolving state that includes IL-10- and TGF-β-associated signaling to connect cell clearance with tissue repair and tolerance (Doran et al., 2020; Shi et al., 2024). This pathway is especially relevant to the ITP because apoptosis of activated lymphocytes or damaged parenchymal cells can remain immunologically quiet when clearance is rapid, whereas defective efferocytosis can convert an initially non-lytic event into persistent inflammation.

By contrast, necroptosis and pyroptosis are membrane-disruptive RCD programs with greater potential to amplify innate immunity. Necroptosis is canonically executed through RIPK3-dependent activation of MLKL, whereas pyroptosis is mediated by gasdermin pore formation downstream of inflammatory caspases or, in some settings, caspase-3/GSDME. These pathways can promote DAMP release and, in pyroptosis, facilitate the release of IL-1β and IL-18 to recruit and activate myeloid and lymphoid cells (Galluzzi et al., 2018; Hirao et al., 2025). Importantly, the susceptibility of hepatocytes to canonical GSDMD-mediated pyroptotic lysis is context-dependent, and some experimental systems have shown gasdermin cleavage without the rapid lytic morphology observed in macrophages; therefore, pyroptosis in liver tissue should be inferred based on mechanistic evidence rather than inflammatory markers alone (Sun et al., 2022).

Ferroptosis is caused by iron-dependent phospholipid peroxidation and a failure of lipid-peroxide detoxification, including GPX4-mediated protection. Due to the liver’s high-metabolism and central-organ functions for iron regulation, this pathway is highly pertinent to ischaemia-reperfusion injury and cancer. Experimental and translational research have linked ferroptosis to liver ischaemia-reperfusion injury, and oxidized lipids and damage signals can promote sterile inflammation (Hirao et al., 2025; Guo et al., 2024). In HCC, ferroptotic pressure is strongly compartment dependent: ferroptosis resistance has been associated with tumor-cell survival, whereas iron accumulation and lipid peroxidation in tumor-infiltrating CD8^+^ T cells may impair effector function and contribute to immune escape (Qin et al., 2025; Chao et al., 2025).

These death categories are not immunological synonyms. Immunogenic cell death (ICD) is a functional outcome in which dying cancer cells promote productive adaptive immunity through coordinated danger signaling, including calreticulin exposure, extracellular ATP, HMGB1 release, and type I interferon-related signals; it cannot be inferred from a single morphology or biomarker (Galluzzi et al., 2020). Moreover, apoptosis, pyroptosis, necroptosis, and ferroptosis can intersect, and autophagy can alter susceptibility to several death programs without necessarily constituting cell death itself (Galluzzi et al., 2018; Hirao et al., 2025). A useful extension of the ITP is therefore a “death-to-immunity axis”: efficiently cleared death tends to favor resolution and tolerance, whereas spatially concentrated lytic or immunogenic death can shift the system toward inflammation or antitumor immunity. Table 1 summarizes the major relationships most relevant to transplantation and HCC.

**TABLE 1:** Major cell-death modalities and their immune consequences in liver transplantation and HCC.

Modality	Core machinery/feature	Typical immune consequence	Relevance to transplantation and HCC
Apoptosis/efferocytosis	Caspase-dependent dismantling; phosphatidylserine recognition; MerTK/TIM4-mediated clearance	Usually non-lytic and pro-resolving when clearance is efficient; defective clearance can lead to secondary necrosis	Supports resolution after tissue injury and can reinforce tolerogenic macrophage programs
Necroptosis	RIPK1/RIPK3/MLKL signaling in canonical contexts	Membrane rupture and DAMP-rich inflammation; effects depend on timing and cellular state	May amplify graft injury; sublethal signaling in hepatocytes can sustain procarcinogenic inflammation
Pyroptosis	Inflammasome/inflammatory caspases-GSDMD or caspase-3-GSDME	Lytic inflammatory death with cytokine and danger-signal release; cell-type dependent	Implicated in hepatic IRI; tumor-cell GSDME pyroptosis can enhance CD8^+^ T-cell responses in HCC models
Ferroptosis	Iron-dependent phospholipid peroxidation; GPX4-centered antioxidant defenses	Oxidized-lipid and DAMP signaling; immune effect depends on which cell undergoes ferroptotic stress	Relevant to hepatic IRI; HCC-cell ferroptosis resistance and CD8^+^ T-cell lipid peroxidation can both favor tumor persistence
Immunogenic cell death	Functional phenotype defined by coordinated danger signaling (e.g., calreticulin, ATP, HMGB1, type I IFN)	Dendritic-cell activation, antigen cross-presentation, and tumor-specific T-cell priming	Provides a framework for converting therapy-induced HCC cell death into productive antitumor immunity; requires functional validation -

## 4 “Beneficial tolerance”: establishment and clinical utilization of the ITP in liver transplantation

In the early period after liver transplantation, the immune milieu is initially shaped by ischemia-reperfusion injury, damage-associated molecular patterns, complement activation, and innate immune-cell activation. It only later transitions toward a long-term balance jointly determined by alloreactive T cells, regulatory cells, and tissue-repair processes (Figure 2) (Que et al., 2023). Spatiotemporal studies in animal transplantation models show that the abundance, localization, and transcriptional states of myeloid and lymphoid cells within the graft change markedly over time, indicating that spontaneous tolerance is a dynamic remodeling process rather than a stable phenotype established immediately after transplantation (Que et al., 2023). During this process, Kupffer cells may either amplify ischemia-reperfusion-associated inflammation or promote tolerance through phagocytosis, IL-10 production, and regulation of T-cell responses; their net effect therefore depends on the phase of injury and the combination of local signals (Zheng et al., 2023). Clinically, this implies that “enhancing tolerance” should not mean indiscriminate suppression of all myeloid responses. Instead, clearance of necrotic tissue and antimicrobial defense should be preserved while cellular states that favor tissue repair and donor-specific hyporesponsiveness are promoted (Zheng et al., 2023).

**FIGURE 2:**
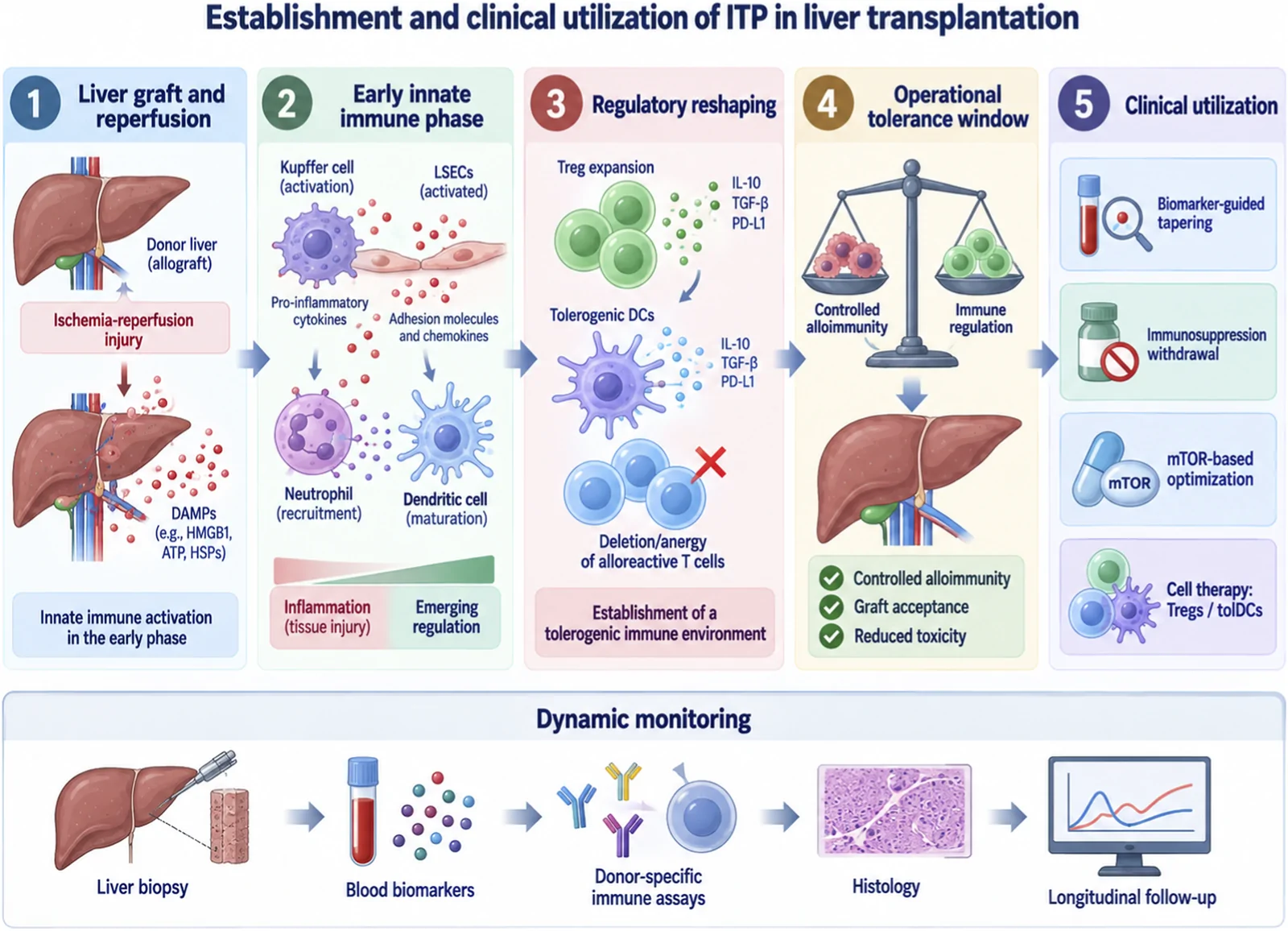
Establishment and clinical utilization of the ITP in liver transplantation.

Identification of operational tolerance is moving from phenotype-based clinical assessment toward stratified management guided by histology and molecular biomarkers (Tanimine et al., 2025; Angelico et al., 2026). A randomized study evaluating the utility of liver-tissue transcriptional biomarkers for guiding immunosuppression withdrawal showed that a single transcriptional signature was not sufficient to replace long-term histological surveillance (Vionnet et al., 2025). Consistent with this view, prospective cohorts have proposed candidate markers such as donor-specific immune senescence, yet the proportion of recipients meeting strict histological criteria for complete operational tolerance is lower than estimates based only on clinical stability (Tanimine et al., 2025). Future tolerance assessment will therefore more likely rely on a composite of peripheral-blood immunophenotyping, donor-reactivity assays, graft transcriptional profiling, and serial histology rather than on a universal single biomarker (Tanimine et al., 2025; Vionnet et al., 2025).

Ischemia-reperfusion injury represents a critical window in which the ITP can be acutely disrupted, because sterile inflammation rapidly activates Kupffer cells, neutrophils, and complement, induces endothelial dysfunction, and amplifies adaptive immunity, thereby increasing the subsequent risk of rejection (Hirao et al., 2022). The therapeutic goal during this phase should not simply be maximal immunosuppression, but rather reduction of tissue injury and danger-signal intensity so that the graft can return more rapidly from an inflammation-dominant state to a microenvironment permissive for tolerance (Hirao et al., 2022).

From a cell-death perspective, hepatic ischemia-reperfusion injury can be viewed as a feed-forward circuit in which oxidative stress and mitochondrial dysfunction initiate parenchymal and endothelial injury, death-associated products activate Kupffer cells and neutrophils, and inflammatory mediators then trigger further tissue damage. Experimental evidence supports roles for ferroptosis and gasdermin-associated pyroptotic signaling in this circuit (Hirao et al., 2025; Guo et al., 2024; Tong et al., 2023), although the contribution of *bona fide* hepatocyte pyroptosis remains model dependent (Sun et al., 2022). This suggests a graft-protective strategy that is conceptually distinct from broad immunosuppression: transiently limiting high-amplitude lytic death and/or improving efficient efferocytic clearance during preservation and early reperfusion, while preserving antimicrobial and alloimmune competence. At present, most death-pathway-targeted approaches remain preclinical and should not be equated with established transplant therapy.

Current immunosuppressive agents affect different components of the ITP in distinct ways. Calcineurin inhibitors (CNIs) effectively restrain T-cell activation but are associated with nephrotoxicity and metabolic adverse effects, whereas mTOR inhibitors have a different spectrum of effects across antiproliferative activity, T-cell differentiation, and oncological risk (Panackel et al., 2022). Tregs are known to be involved in the adaptive hyporesponsiveness following transplantation, so efforts to expand, stabilize and monitor the function of Tregs have been proposed as therapeutic strategies (Han and Park, 2024). Tolerogenic dendritic cells (DCs) can also induce antigen-specific hyporesponsiveness by reducing costimulation, releasing immunoregulatory factors and promoting Tregs, and have the theoretical advantage of avoiding non-specific systemic immunosuppression (Hadjiyannis and Thomson, 2024). Approaches such as adoptive Treg transfer and tolerogenic DCs are now relatively well-defined in terms of clinical development; however, their efficacy, manufacturing consistency, infection risk and long-term safety all require further prospective studies (Zhou et al., 2024). mTOR inhibitors have been studied in patients with HCC to reduce CNIs and provide some anti-cancer effects; however, due to variations in patient selection, dosage and endpoints among the studies, it is not clear which ones are most suitable (Todeschini et al., 2023).

## 5 “Detrimental tolerance”: spatial reprogramming of the ITP in hepatocellular carcinoma

If the goal of transplant tolerance is to guide the ITP into a window that “protects the graft while preserving host defence”, HCC has shown that tumours can drive the same programme towards persistent immunosuppression and effector T-cell dysfunction (Zheng et al., 2017; Zhang et al., 2019). Early single-cell studies have shown significant clonal and functional heterogeneity in HCC-infiltrating T cells. The distribution of exhausted CD8^+^ T cells, Tregs, and different memory/effector subsets showed that anti-tumor immunity is not solely determined by the quantity of immune infiltration but also by the cellular state and clonal architecture (Zheng et al., 2017). Subsequent large-scale single-cell atlases have further revealed that myeloid cells, T cells and dendritic cells form intricate interaction networks in HCC, and that the tumor microenvironment of tumor tissue and adjacent liver exhibits marked differences (Zhang et al., 2019). Tumor vasculature is not a passive tube; hepatocellular carcinoma (HCC) can induce oncofetal-like endothelial reprogramming that supports immunosuppressive macrophages and links vascular abnormalities to myeloid immunosuppression (Sharma et al., 2020). Therefore, ITP reprogramming in HCC is composed of at least three interconnected parts: impaired effector T-cell function, an accumulation of suppressive myeloid cells, and a physical-biochemical barrier formed by abnormal stroma and vasculature (Zheng et al., 2017; Zhang et al., 2019; Sharma et al., 2020).

Spatial omics has organised the cell ‘inventory’ into anatomically organised immune structures (Liu et al., 2023; Wu et al., 2023). Stereo-seq shows an approximately 500-µm invasive zone at the margin of HCC. It was proposed that damaged hepatocytes recruit and rearrange macrophages via the CXCL6- and SAA1/2-related axis, forming a boundary niche that promotes local immunosuppression and tumour progression (Wu et al., 2023). Therefore, the tumour margin is not only the interface of normal liver tissue and cancer cells but may also act as a biological “first line of defence” to limit microscopic invasion, recurrence and local immune evasion (Wu et al., 2023). At the stromal level, POSTN-positive CAFs can promote the accumulation of SPP1-positive macrophages through mechanisms including IL-6/STAT3 signaling and are associated with reduced sensitivity to immunotherapy (Wang et al., 2024). Other research has shown that DAB2-positive macrophages support FAP-positive fibroblasts to form a tumor barrier and are associated with a poor prognosis; therefore, a bidirectional reinforcement loop between myeloid cells and fibroblasts has been further established (Long et al., 2024). Spatial proteomics has also identified vimentin-high macrophages with immunosuppressive properties, and conventional classifications of macrophages based on a small number of surface markers fail to capture the actual functional states in HCC (Qiu et al., 2024). Independent integration of single-cell and transcriptomic analyses has also identified SPP1-positive macrophages as a primary immunosuppressive subpopulation, and their abundance is related to immunotherapy response and prognosis (Jin et al., 2024). Collectively, the above data support a central conclusion: “detrimental tolerance” in HCC is not a general amplification of physiological ITP, but rather a tumor-driven, spatially selective reprogramming and structural fixation of the ITP at specific anatomical sites (Liu et al., 2023; Wu et al., 2023; Wang et al., 2024; Long et al., 2024; Qiu et al., 2024; Jin et al., 2024).

B cells and tertiary lymphoid structures (TLSs) further demonstrate the dual nature of ITP, as B cells can regulate the immune system but may also support antigen presentation and T-cell activation in mature TLSs to promote local anti-tumor responses (Nie et al., 2022). The number, maturation stage and location of TLSs in HCC are related to the composition of immune cells; thus, merely knowing that TLSs are present or absent does not reveal any functional differences (Nie et al., 2022). Human tissue studies before and after immunotherapy show that treatment responses can be accompanied by divergent TLS morphologies and cellular compositions, suggesting that TLSs may serve both as markers of immune activity and as dynamic products of treatment-induced remodeling (Shu et al., 2024). Portal vein tumor thrombus (PVTT) represents another specialized spatial niche. Single-cell studies have found that its myeloid composition and immunosuppressive features are not identical to those of the primary tumor and have implicated C5aR-associated macrophages in the establishment of the intravascular growth environment (Zhou et al., 2025a)^.^ Accordingly, for HCC with vascular invasion, future immune classification should not rely exclusively on biopsies of the primary tumor but should also consider potential spatial discordance between venous tumor thrombi and invasive margins (Wu et al., 2023; Zhou et al., 2025a).

Beyond the extrinsic microenvironment, tumor-cell-intrinsic signaling can actively determine ITP intensity (Zheng et al., 2017; Ruiz de Galarreta et al., 2019). Activation of the beta-catenin pathway can impair dendritic-cell recruitment and promote T-cell exclusion, and both model systems and human evidence associate this state with resistance to anti-PD-1 therapy, thereby establishing a direct link between tumor genotype, an immune-cold microenvironment, and treatment resistance (Ruiz de Galarreta et al., 2019).

RCD adds another layer of context dependence to HCC biology. In a preclinical mouse model, sublethal RIPK1/RIPK3/MLKL-associated signaling was reported to sustain chemokine production and promote procarcinogenic macrophage states, illustrating that activation of a death pathway does not necessarily translate into tumor-cell clearance (Vucur et al., 2023). Conversely, oxaliplatin-induced caspase-3/GSDME pyroptosis has been reported to enhance CD8^+^ T-cell infiltration and cytotoxicity in HCC models, providing a mechanistic example of therapy-induced inflammatory death promoting antitumor immunity (Deng et al., 2025). Ferroptosis is also cell-type specific: tumour-cell ferroptosis resistance has been associated with HCC growth and restricted T-cell recruitment, whereas iron accumulation and lipid peroxidation in tumour-infiltrating CD8^+^ T cells may directly impair effector function (Qin et al., 2025; Chao et al., 2025). Based on the above results, death-targeting strategies should be designed according to the type and location of cells, rather than simply aiming to increase cell death.

Clinically, PD-L1 blockade combined with VEGF inhibition has reshaped first-line treatment for advanced HCC, while dual CTLA-4/PD-L1 immunotherapy has also shown that simultaneous modulation of distinct immune nodes can yield durable survival benefit (Finn et al., 2020; Abou-Alfa et al., 2022). A further phase III study demonstrated that PD-1 inhibition combined with antiangiogenic targeted therapy can significantly improve survival in unresectable HCC, supporting a clinically exploitable synergy between vascular normalization and immune restoration (Qin et al., 2023). Recent randomized data for a first-line dual-checkpoint regimen further indicate that combined intervention at the T-cell priming and effector phases can outperform conventional tyrosine kinase inhibitors; nevertheless, benefit remains confined to a subset of patients, underscoring that myeloid, stromal, and tumor-intrinsic resistance mechanisms remain incompletely addressed (Yau et al., 2025). The next phase of HCC immunotherapy should therefore not consist of indefinitely stacking checkpoint agents, but of mechanism-guided combinations selected according to the specific dysregulated nodes of the ITP (Liu et al., 2023; Wang et al., 2024; Ruiz de Galarreta et al., 2019; Abou-Alfa et al., 2022; Qin et al., 2023; Yau et al., 2025).

## 6 A double-edged sword: systematic comparison of the ITP in liver transplantation and HCC

Placing liver transplantation and HCC within the same framework reveals that many ITP components operate through related mechanisms yet have opposite clinical implications (Figure 3) (Gottwick et al., 2022; Zheng et al., 2023; Zheng et al., 2017). In the early post-transplant period, Kupffer cells and related tumor-associated macrophage populations can promote tissue repair by clearing damage-associated material and producing regulatory mediators; in HCC, however, macrophages dominated by SPP1, or other suppressive programs can cooperate with CAFs, abnormal endothelium, and exhausted T cells to form immune-exclusion structures (Zheng et al., 2023; Sharma et al., 2020; Wang et al., 2024; Qiu et al., 2024; Jin et al., 2024). Physiologically and in transplantation, LSECs suppress excessive T-cell activation by presenting low-costimulatory antigens; otherwise, in tumours, oncofetal endothelial reprogramming and abnormal vascular architecture can prevent effective lymphocyte entry and promote the survival of suppressive myeloid cells (Shetty et al., 2018; Sharma et al., 2020). Hepatic stellate cells participate in matrix deposition during tissue repair, but after prolonged activation, they can transform into immune-regulatory fibroblasts that secrete factors and collagen-rich matrices to facilitate the tumour’s utilisation of a “repair program” for growth promotion (Wang et al., 2024; Long et al., 2024). Tregs help to suppress alloreactivity and establish donor-specific hyporesponsiveness after transplantation, and persistent enrichment of Tregs in HCC can consume growth factors, inhibit effector T cells, and reduce the effect of immune checkpoint blockade (Han and Park, 2024; Zheng et al., 2017; Zhang et al., 2019). Based on the above comparison, the design of therapy should focus on cellular “states” rather than simply cellular “names”, because the same lineage can have different biological functions in different tissues (Araujo David et al., 2026; Zheng et al., 2023; Qiu et al., 2024).

**FIGURE 3:**
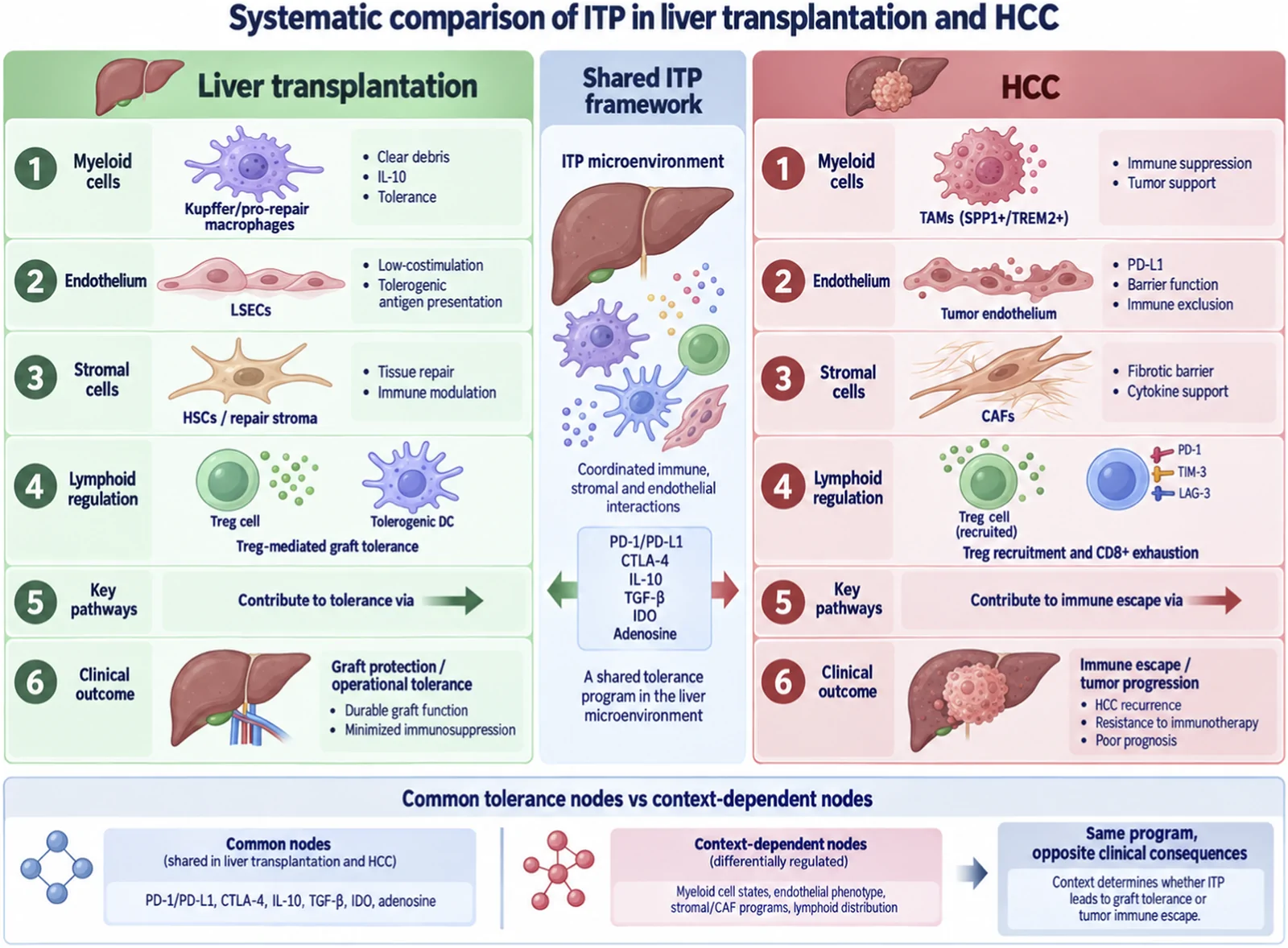
Systematic comparison of the ITP in liver transplantation and HCC.

Molecular pathways also have context-dependent directional variations (Gottwick et al., 2022; Finn et al., 2020). PD-1/PD-L1 and CTLA-4 can lower the activation threshold of alloreactive T cells in the transplant setting and, in theory, promote the development of hyporesponsiveness; therefore, to restore tumor-specific T-cell function for HCC treatment, these signals need to be blocked (Gottwick et al., 2022; Finn et al., 2020; Abou-Alfa et al., 2022; Gupta et al., 2019). TGF-beta, IL-10, IDO and adenosine pathways are involved in tissue repair and the regulation of immunopathology; however, chronic activation of these pathways can suppress T cells, reprogram myeloid cells and increase extracellular matrix accumulation, thereby promoting tumour immune evasion (Kubes and Jenne, 2018; Gottwick et al., 2022; Wang et al., 2024). Therefore, the two types of ITP nodes can be divided into “shared tolerance nodes”, such as PD-1/PD-L1 and Treg stability, which have relatively consistent biological directions in all environments but different therapeutic goals; and “context-dependent nodes”, such as the SAA1/2-CXCL6 axis in the invasive zone, POSTN-positive CAFs, or specific tumor endothelial states, which only occur under certain pathological conditions (Wu et al., 2023; Wang et al., 2024). In the context of precision therapy, selecting context-dependent nodes may be safer than systemically disrupting shared tolerance mechanisms because, in principle, it could reduce the damage to antimicrobial defence, graft homeostasis and immune tolerance in normal tissues (Liu et al., 2023; Wu et al., 2023; Wang et al., 2024; Long et al., 2024).

Given the above analysis, the factor of clinical relevance is a person’s position on the “tolerance continuum” (Gottwick et al., 2022; Tanimine et al., 2025). A stable liver-transplant recipient does not require maximum immunosuppression; instead, it is desirable to reduce the toxicity of long-term drug administration while preventing rejection and, when histological safety can be verified, moving towards an operational-tolerance range (Tanimine et al., 2025; Angelico et al., 2026; Vionnet et al., 2025). For nontransplanted patients with HCC, the goal is instead to selectively dismantle local tumor-associated immunosuppression while preserving the anti-inflammatory and reparative functions required in nontumorous liver tissue (Liu et al., 2023; Wu et al., 2023; Wang et al., 2024; Long et al., 2024; Qiu et al., 2024; Jin et al., 2024). In patients with recurrent HCC after liver transplantation, these two objectives coexist; consequently, any strategy that unidirectionally intensifies either immune activation or immune suppression may impose unacceptable costs (Kayali et al., 2023; Dituri et al., 2026). This is precisely why “precision immune homeostasis” is a more appropriate therapeutic framework for this population than the binary concept of “immune activation versus immune suppression” (Gottwick et al., 2022; Kayali et al., 2023; Dituri et al., 2026).

## 7 Clinical crossroads: HCC recurrence after liver transplantation

All levels of the clinical evidence considered in this section should not be treated equally. Randomised controlled trials provide high-quality evidence of the effectiveness of treatment but often do not include transplant recipients; thus, their application to the post-transplant period is limited (Ayaz-Shah et al., 2018). Prospective cohorts and individual-patient-data meta-analyses are more direct forms of observation. Retrospective multicenter cohorts, pooled analyses and systematic reviews of heterogeneous cases are currently the main sources of information on immune checkpoint inhibitor (ICI) use after liver transplantation; they are prone to selection bias, confounding factors, variable immunosuppression, and incomplete reporting (Aceituno et al., 2026; Liu et al., 2025; Rezaee-Zavareh et al., 2025). Case series and case reports can identify serious adverse events, such as acute rejection, but not reliably determine the frequency or conduct comparative safety analyses (Friend et al., 2017). Preclinical and mechanistic studies have provided biological plausibility for the role of the PD-1/PD-L1 axis in preserving peripheral graft tolerance, but have not independently supported clinical application (Morita et al., 2010). Therefore, the following statements distinguish between evidence obtained from randomised data, observational studies, case reports and experimental models, and do not put forward any particular safety ranking or routine treatment algorithm in the absence of high-level evidence.

HCC recurrence after liver transplantation is the most clinically challenging manifestation of ITP duality, because patients require sufficient immunosuppression to prevent graft rejection while also needing restoration of tumor immune surveillance to control recurrent disease (Kayali et al., 2023; Dituri et al., 2026). In HCC patients who receive ICIs before transplantation, an individual-patient-data meta-analysis showed that post-transplant rejection risk is closely related to the interval between immunotherapy and transplantation; a longer washout period may reduce the probability of rejection, although current evidence derives largely from retrospective cases and highly heterogeneous cohorts (Rezaee-Zavareh et al., 2025). These findings indicate that ICI effects do not disappear immediately when treatment is stopped. Persistent immunological changes, including receptor occupancy, activated T-cell clones, and tissue memory, may extend across the transplant surgical window (Rezaee-Zavareh et al., 2025). Therefore, for patients entering the transplant waiting list after neoadjuvant or bridging ICI therapy, an “immunological washout interval” should be incorporated into organ-allocation and perioperative risk assessment rather than allowing surgical timing to be determined solely by radiographic tumor response (Rezaee-Zavareh et al., 2025).

The risk is even more direct when ICIs are reintroduced after post-transplant recurrence (Kayali et al., 2023; Dituri et al., 2026). Systematic reviews and pooled analyses show that clinically significant acute rejection can occur when transplant recipients with malignancy receive ICIs, and when rejection leads to graft failure the consequences may be fatal (Kayali et al., 2023). The latest systematic evidence specifically addressing HCC after liver transplantation likewise suggests that ICIs can achieve tumor control in a minority of patients, but rejection is not uncommon and severe rejection may rapidly progress to graft failure; standard treatment pathways for advanced HCC in nontransplant patients therefore cannot be applied directly (Kahramangil et al., 2026). Case reports and small cohorts further suggest that the PD-1/PD-L1 axis may play an important role in maintaining peripheral graft tolerance, providing a biological explanation for rejection after blockade of this pathway (Jiang et al., 2023). Available evidence is insufficient to establish a definitive safety ranking among checkpoint agents. The claim that CTLA-4 blockade is necessarily safer than PD-1 blockade still lacks high-quality randomized evidence; clinical choice should therefore be framed as an individualized risk-benefit assessment rather than a fixed hierarchy (Kahramangil et al., 2026; Jiang et al., 2023; Xu et al., 2018; Oliveira et al., 2024). Accordingly, when ICIs are considered for post-transplant recurrence, treatment should be concentrated in experienced multidisciplinary centers and accompanied, after fully informed consent, by intensive monitoring of liver function, immunosuppressant concentrations, and rejection (Kahramangil et al., 2026; Jiang et al., 2023; Marrone et al., 2023).

The immunosuppressive regimen itself is also part of recurrence management (Todeschini et al., 2023; Marrone et al., 2023). Prolonged high-intensity calcineurin inhibition in HCC transplant recipients may raise oncological concerns, whereas in appropriately selected patients reducing CNI exposure and introducing an mTOR inhibitor is considered a potential strategy for balancing graft protection with antiproliferative effects (Todeschini et al., 2023; Marrone et al., 2023). Systematic evaluation of randomized trials suggests that mTOR inhibitor-based regimens may confer oncological or survival advantages in some HCC transplant populations, although the magnitude of benefit is influenced by transplant indication, tumor risk, timing of therapy, and regimen heterogeneity (Aloun et al., 2023). Thus, after recurrence, the most reasonable approach is generally not abrupt withdrawal of immunosuppression but stepwise optimization according to prior rejection, graft function, tumor burden, and available anticancer therapies (Marrone et al., 2023; Aloun et al., 2023). From an ITP perspective, this process effectively moves the patient from “drug-induced deep tolerance” toward “minimum effective graft tolerance” while avoiding the threshold at which acute rejection is triggered (Kayali et al., 2023; Marrone et al., 2023; Aloun et al., 2023).

## 8 Translational pathway: from immune binary thinking to precision immune homeostasis

Based on the evidence reviewed above, we propose that “precision immune homeostasis” should comprise three sequential steps: identifying the patient’s clinical context, determining the dominant ITP nodes, and continually recalibrating intervention intensity through dynamic monitoring (Gottwick et al., 2022; Dituri et al., 2026). In stable liver-transplant recipients without malignancy, the primary objective is to enhance donor-specific rather than systemic tolerance; therefore, Tregs, tolerogenic DCs, antigen-specific delivery, and local immune modulation are mechanistically more coherent than chronically intensifying broad-spectrum immunosuppression (Han and Park, 2024; Hadjiyannis and Thomson, 2024). Liver-targeted nanodelivery platforms provide a new engineering tool for this strategy, because drugs or tolerogenic antigens that preferentially accumulate in specific hepatic cell populations may promote local immune tolerance while reducing systemic exposure (Kusumoputro et al., 2023). However, such biomaterial and cellular approaches still need to address manufacturing standardization, long-term safety, risks of infection and malignancy, and whether true donor-specific immunological memory can be established (Zhou et al., 2024; Kusumoputro et al., 2023).

For patients with HCC, the objective of precision immune homeostasis is the opposite: to interrupt tumor-specific ITP reprogramming while preserving as much physiological tolerance in the normal liver as possible (Gottwick et al., 2022; Liu et al., 2023; Zhou L. et al., 2025). Spatial and single-cell profiling may eventually enable the classification of patients into immune ecotypes characterized by T-cell exhaustion, myeloid suppression, a CAF barrier, abnormal vasculature, or beta-catenin-associated immune exclusion; however, these methods are currently limited to research tools and have not been prospectively validated for routine treatment selection. Their value lies in identifying cellular states that can inform the development of mechanism-guided, dual-checkpoint, antiangiogenic, myeloid-reprogramming or stromal-targeting strategies in future clinical studies (Sharma et al., 2020; Liu et al., 2023; Wu et al., 2023; Wang et al., 2024; Long et al., 2024; Qiu et al., 2024; Jin et al., 2024; Ruiz de Galarreta et al., 2019; Abou-Alfa et al., 2022; Qin et al., 2023; Yau et al., 2025; Kayali et al., 2023; Salié et al., 2025; Wang et al., 2023). For example, in “barrier-type” tumors enriched for both POSTN-positive CAFs and SPP1-positive macrophages, simply intensifying PD-1 blockade may be insufficient to permit effector T cells to enter the tumor core; the CAF-macrophage axis may therefore represent a more direct combination target (Liu et al., 2023; Wang et al., 2024; Jin et al., 2024). In beta-catenin-driven immune-excluded tumors, priority should instead be given to restoring dendritic-cell recruitment and T-cell entry rather than attributing primary resistance simply to “insufficient PD-1 dosing” (Ruiz de Galarreta et al., 2019). TLS maturity, invasive-zone status, and the PVTT immune niche may provide additional dimensions for spatial immune classification, extending the traditional single-site biopsy into a multiregional risk map (Wu et al., 2023; Nie et al., 2022; Shu et al., 2024; Zhou et al., 2025a.

A third proposed component is longitudinal monitoring, which has yet to be prospectively validated in HCC and post-transplant settings (Tanimine et al., 2025; Vionnet et al., 2025). Recently, prospective studies have examined the long-term changes in immune biomarkers after immunosuppression withdrawal and proposed that tracking the trajectory of these biomarkers during dose reduction may be more informative than seeking a single static “tolerance signature” (López-Díaz et al., 2025). A similar approach has been proposed for HCC, and in this study, circulating tumour DNA, peripheral immune-cell profiles, cytokines, imaging, and if clinically appropriate, repeated tissue sampling can be incorporated into longitudinal studies to determine whether a patient has transitioned from reversible immunosuppression to stable immune escape or acquired resistance; however, whether such multimodal monitoring improves treatment selection or clinical outcomes has not been determined (Zhang et al., 2019; Liu et al., 2023; Jin et al., 2024). To address the issue of post-transplant recurrence, a “dual-endpoint” observation system has been proposed to assess both the probability of tumour control and the probability of rejection simultaneously, rather than relying solely on objective tumour response data (Kahramangil et al., 2026; Jiang et al., 2023; Marrone et al., 2023). AI and multi-omics models may eventually facilitate the integration of longitudinal, multi-tissue, and high-dimensional data into interpretable risk-stratification tools, but their clinical utility in transplant oncology has not been prospectively verified (Tanimine et al., 2025; Vionnet et al., 2025; López-Díaz et al., 2025).

RCD profiling can provide an additional type of data for this dynamic monitoring system, but at present, it is expected to be a research direction rather than a standard clinical decision-support tool. Candidate readouts include gasdermin cleavage for pyroptotic signalling, phosphorylated MLKL for necroptotic pathway activation, GPX4 status and lipid-peroxidation products for ferroptotic stress, and extracellular HMGB1 or related DAMPs as indicators of immunologically active tissue injury (Galluzzi et al., 2018; Galluzzi et al., 2020; Hirao et al., 2025). As these markers can be produced by various types of cells and are indicative of pathway activation without cell death, they should not be used alone as surrogate endpoints but rather in combination with histology, spatial information and immune-cell phenotypes.

Importantly, the same immunotherapy regimen is not suitable for all stages of the disease following successful immunotherapy (Finn et al., 2020; Yopp et al., 2026). Even for resectable HCC in the adjuvant setting, an early positive signal cannot be automatically considered beneficial in the long run; as shown by updated follow-up data from IMbrave050, the initial recurrence-free survival advantage was not maintained, and both disease stage and the length of follow-up affect the evaluation of immunotherapy (Yopp et al., 2026). Transplant oncology is a typical example: the immune thresholds at the time of transplantation, during the stable post-transplantation period and after disease relapse are not the same; therefore, safety or efficacy observed at any one time cannot be directly generalised to other circumstances (Dituri et al., 2026; Rezaee-Zavareh et al., 2025; Kahramangil et al., 2026). Future trials should adopt mechanism-stratified designs, record graft PD-L1 expression, previous rejection status, CNI/mTOR inhibitor exposure, tumor spatial immune phenotype and peripheral T-cell clonal features at enrollment, and preset a joint endpoint of “tumor control-graft injury” (Dituri et al., 2026; Kahramangil et al., 2026; López-Díaz et al., 2025). Such studies would be required to move the ITP from an explanatory framework toward an implementable, evidence-based clinical decision system (Gottwick et al., 2022; Dituri et al., 2026).

## 9 Conclusions and perspectives

Liver immune tolerance is not a single protective mode but a tissue-level immunoregulatory network that can be altered in all kinds of living and diseased states (Kubes and Jenne, 2018; Shetty et al., 2018; Araujo David et al., 2026; Gottwick et al., 2022). Regulated cell death and the clearance of dying cells are part of this network: apoptosis combined with efficient efferocytosis can enhance resolution, while necroptosis, pyroptosis, ferroptosis, or immunogenic tumour-cell death may redirect local immunity towards sterile inflammation or anti-tumour activation depending on the cell type and spatial location (Galluzzi et al., 2018; Doran et al., 2020; Shi et al., 2024; Galluzzi et al., 2020; Hirao et al., 2025; Sun et al., 2022; Guo et al., 2024; Qin et al., 2025; Chao et al., 2025; Vucur et al., 2023; Deng et al., 2025). Optimally enhance and precisely identify this network in liver transplantation to reduce alloreactivity, lower the extended requirement for immunosuppression, and create an environment favourable to organ function; otherwise, premature or unjustified discontinuation of immunosuppression can still lead to delayed graft failure (Tanimine et al., 2025; Angelico et al., 2026; Vionnet et al., 2025). In HCC, tumors remodel macrophages, endothelium, CAFs, T cells, TLSs, and related structures, converting a program originally used to limit hepatic immunopathology into an advantage for immune escape, while spatial omics has localized this process to defined niches such as invasive zones and tumor immune barriers (Zheng et al., 2017; Zhang et al., 2019; Sharma et al., 2020; Liu et al., 2023; Wu et al., 2023; Wang et al., 2024; Long et al., 2024; Qiu et al., 2024; Jin et al., 2024; Nie et al., 2022; Shu et al., 2024). In HCC recurrence after liver transplantation, these two logics collide directly, making antitumor benefit and graft-rejection risk simultaneous endpoints that must be evaluated together (Kayali et al., 2023; Dituri et al., 2026; Kahramangil et al., 2026). Future clinical strategies should therefore avoid pursuing ever-stronger immune activation or immunosuppression as ends in themselves. Instead, context identification and node selection, together with longitudinal monitoring as it becomes validated, may help place each patient within an immune-homeostatic range appropriate to the current disease stage (Han and Park, 2024; Hadjiyannis and Thomson, 2024; Zhou et al., 2024; López-Díaz et al., 2025). Single-cell and spatial omics, longitudinal biomarkers, liver-targeted delivery, and cellular therapy will provide the technical foundation for this goal; however, whether these advances ultimately improve outcomes will depend on prospective, mechanism-stratified clinical trials that evaluate both tumor and graft endpoints (Zhou et al., 2024; Wu et al., 2023; López-Díaz et al., 2025; Chen et al., 2025). Therefore, the “double-edged sword” nature of the ITP should not be viewed as a problem to be solved, but rather as an intrinsic characteristic of cells that can be synergistically harnessed by transplantation medicine and tumour immunology to achieve precise regulation and clinical benefits (Gottwick et al., 2022; Dituri et al., 2026; Kahramangil et al., 2026).
